# Analysis of clinical characteristics and genetic testing in patients with acute fatty liver of pregnancy: a retrospective study

**DOI:** 10.1186/s12884-021-04095-8

**Published:** 2021-09-08

**Authors:** Yixian Shi, Haicong Wu, Xiaoling Zhou, Qingling Xu, Liqing Zheng, Dongliang Li, Lvfeng Yao

**Affiliations:** 1 Department of Hepatology, Mengchao Hepatobiliary Hospital, Fujian Medical University, Fuzhou, 350025 Fujian China; 2 Department of Hepatobiliary Medicine, 900th Hospital of PLA, Fuzhou, 350025 Fujian China; 3 Fuzong Clinical College, Fujian Medical University, Fuzhou, 350025 Fujian China

**Keywords:** Acute fatty liver of pregnancy, Clinical characteristics, Genetic testing, Whole-exome sequencing

## Abstract

**Background:**

Acute fatty liver of pregnancy (AFLP) is an acute, rare and potentially lethal disease typically occurring in the third trimester of pregnancy. So far, there is no effective means of prevention. Therefore, in this study, we retrospectively analyzed the clinical features of AFLP patients for a better understanding. Meanwhile, for the first time, the genetic background associated with the onset of AFLP was discussed by high-throughput sequencing, hoping to provide evidence for genetic counseling and prenatal diagnosis of AFLP.

**Methods:**

Thirteen AFLP patients admitted to our hospital and other hospital from March 2012 to February 2020 were selected. Clinical data about general condition, laboratory test, liver biopsy and the prognosis of mother and fetus were collected for retrospective analysis. In addition, the peripheral blood of five patients with AFLP and one newborn infant of his mother with AFLP was sequenced with whole-exome sequencing and gene mutation was analyzed by bioinformatics methods.

**Results:**

The initial symptoms of AFLP varied differently, with jaundice (9/13, 69%), fatigue (8/13, 62%) and nausea and vomiting (6/13, 46%) being the most common. Moreover, the main maternal complications were coagulopathy (13/13, 100%), followed by acute renal dysfunction (10/13, 77%). Raised serum bilirubin, transaminases and uric acid were found in all patients (100%), hypoglycemia was found in six patients (46%) and fatty liver on ultrasound was seen in five patients (5/12, 42%). One (7%) maternal death occurred and all neonates survived delivery. In addition, to our surprise, whole-exome sequencing showed that no gene mutation in related enzymes involved in fatty acid metabolism was noted in the pregnant women and children receiving genetic testing.

**Conclusions:**

Early visit, early detection, early termination of pregnancy and multidisciplinary comprehensive treatment are the key factors to improve the prognosis of AFLP patients and their newborn infants. Furthermore, although limited size of study, to our knowledge, this report is the first to present the lack of common mutation involved in fatty acid oxidation in Chinese patients with AFLP via whole-exome sequencing. Thus, further studies are needed with larger and more varied samples to validate the conclusion.

**Supplementary Information:**

The online version contains supplementary material available at 10.1186/s12884-021-04095-8.

## Background

Acute fatty liver of pregnancy (AFLP) is a relatively rare but potentially fatal obstetric emergency. Epidemiological studies provide the incidence for AFLP of 1 per 7000 to 15,000 deliveries [1–3]. With advances in supportive obstetric care, the maternal and fetal mortality rates have declined from 80 to 85% to 7–18% and from 50% to 9–23% respectively [4–6]. However, extremely low incidence of AFLP in combination with the fact that it is an interdisciplinary subject between liver disease and obstetrics and gynecology inevitably leads to insufficient disease knowledge, which easily causes a missed diagnosis and misdiagnosis. Hence, to maintain keen vigilance of clinical manifestations of AFLP is of vital importance. Women in the third trimester of pregnancy presented with digestive symptoms such as vomiting, nausea, abdominal pain, encephalopathy and jaundice should be especially attentioned. Laboratory and imageology studies reveal that AFLP is mainly characterized by hepatic and renal dysfunction, coagulopathy and bright liver [7]. In order to provide a valuable theoretical basis for the treatment of AFLP, we collected, analyzed and summarized 13 local cases about AFLP admitted to our hospital and other hospital during the past 8 years. In addition, most studies regarding AFLP reported so far in China were just case reports or analysis of clinical data. It’s been reported that the development of AFLP is related to genetic mutations in western people. However, no genetic testing or etiological studies have been performed in China. The present study is the first to perform high-throughput sequencing, hoping to explore the genetic background related to the onset of AFLP in Chinese patients at the genetic level, which could provide clues for clinical diagnosis, genetic counseling and prenatal diagnosis of AFLP.

## Methods

### Study population and ethical considerations

Thirteen AFLP patients admitted to Mengchao Hepatobiliary Hospital, Fujian Medical University and 900th Hospital of PLA Joint Logistics Support Force from March 2012 to February 2020 were selected. This study was approved by the Research Ethics Committee of Mengchao Hepatobiliary Hospital, Fujian Medical University and 900th Hospital of PLA. Informed consent was obtained from all individual participants included in the study.

Patients were enrolled in the study if they met the Swansea criteria, a diagnostic tool for AFLP [1, 2]. After excluding other causes, AFLP can be diagnosed in a patient who fulfilled at least 6 out of the 15 criteria: (1) vomiting; (2) abdominal pain; (3) polydipsia/polyuria; (4) hepatic encephalopathy; (5) bilirubin> 14 μmol/L; (6) hypoglycemia< 4 mmol/L; (7) elevated uric acid> 340 μmol/L; (8) white blood cell count> 11 × 10^9^/L; (9) ascites; (10) elevated transaminase (> 42 U/L); (11) elevated ammonia (> 47 μmol/L); (12) acute renal injury (Cr > 150 μmol/L); (13) coagulopathy (PT > 14 s, or APTT> 34 s); (14) “bright liver” on ultrasound; (15) microvesicular steatosis on liver biopsy.

### Observation indicators

The general information, clinical manifestations, laboratory indicators, abdominal ultrasound, mode of delivery, fetal conditions, treatment measures, length of hospital stay, and maternal and infant prognosis of 13 AFLP patients were analyzed retrospectively and detailly recorded in Additional file 1 and Additional file 2.

### Liver biopsy and histological assessment of AFLP

Percutaneous hepatic biopsy was performed under the guidance of ultrasonography on postnatal day 16 or day 23. Liver biopsy specimens were fixed with 10% neutral formaldehyde, paraffin-embedded, and sliced into 4-μm-thick sections. Histological evaluation was performed according to the conventional protocol by two pathologists blinded to the experiment.

### Whole-exome sequencing

Whole-exome sequencing (WES) was performed by Running Gene Inc. (Beijing, China) to discover the causal gene. Peripheral blood samples from 5 probands and 1 kid were collected. According to the manufacturer’s instructions, DNA samples were isolated using a DNA Isolation Kit (Bioteke, AU1802), qualified by a Qubit dsDNA HS Assay Kit (Invitrogen, Q32851), fragmented into 200–300 bp length by Covaris Acoustic System (Covaris, Woburn, USA) and then prepared with a KAPA Library Preparation Kit (Kapa Biosystems, KR0453). Hybridization of the prepared libraries to the capture probes and removal of non-hybridized DNA fragments were carried out according to the Agilent SureSelectXT2 Target Enrichment System (Agilent, CA, USA). The final products were sequenced as paired-end 150-bp reads on the Illumina HiSeq X platform. The mutations that affected the encoding of amino acids in the exonic regions or in the splice sites including missense, frameshift, stoploss, stopgain and splicing variants were filtered and annotated using GATK variant filtration and ANNOVAR in additional file 3. The potential impacts of the variants were predicted by SIFT, Polyphen-2 and Mutation Taster programs. Variant pathogenicity was assessed according to the American College of Medical Genetics and Genomics (ACMG) guidelines [8].

### Data analysis

Statistical analysis was conducted using SPSS software version 17.0. Data in normal distribution were displayed as mean ± standard deviation (SD). The counting data were expressed as percentage or rate.

## Results

### General information of the AFLP patients in our study

A retrospective analysis was performed on 13 patients with final clinical diagnosis of AFLP admitted from March 2012 to February 2020, ranging from 23 years old to 39 years old, with an average age of 29.2 years. There were 5 cases of multipara and 8 cases of primipara. The onset time ranged from 32 to 38 + 6 weeks, with an average time of 34 + 5 weeks. Among them, there were 4 cases (31%) with gestational weeks less than 34 weeks, 7 cases (54%) with 34 ~ 36 + 6 weeks, and 2 cases (15%) with 37 weeks or more. Visibly, this kind of disease is concentrated in the third trimester. The patients’ visit time is generally 0–30 days after the onset and the diagnosis time is 0–2 days after the visit. All pregnancies were terminated within 24 h of definitive diagnosis. Two pregnant women (case 1 and case 2) were accompanied by serious coagulopathy and DIC due to delayed care seeking, among which 1 pregnant woman died. There were 11 cases of cesarean section and 2 cases of vaginal delivery. Above details are given in Table 1 and Additional file 1.

**Table 1:** The overall summary of clinical features of AFLP patients (*n* = 13)

	Number of cases	Percentage (%)
Clinical symptoms		
fatigue	8	62
jaundice	9	69
nausea and vomiting	6	46
headache	2	15
abdominal pain	1	7
Comorbidities	3	23
Onset time		
< 34 weeks	4	31
34 ~ 36 + 6 weeks	7	54
> 37 weeks	2	15
Gravida		
Primigravida	8	62
Multigravida	5	38
Delivery mode		
caesarean section	11	85
vaginal delivery	2	15
Pregnant outcome		
maternal survival	12	92
maternal death	1	7
perinatal survival	13	100
Fetal sex		
male	8	62
female	5	38
Fatty liver on ultrasound (*n* = 12)	5	42
Liver biopsy	2	15

AFLP: Acute fatty liver of pregnancy

### Clinical manifestations of the 13 AFLP patients

Among the 13 patients, the initial symptoms of AFLP varied differently, with jaundice (9/13, 69%), fatigue (8/13, 62%) and nausea and vomiting (6/13, 46%) being the most common. Besides, three pregnant women (23%) were combined with HELLP syndrome or preeclampsia (see Table 1 and Additional file 1 for details). The relevant laboratory examination results of 13 AFLP patients were shown in Table 2 and Additional file 2 (the recorded values are the maximum outliers from the onset to the outcome of the disease). Liver damage occurred in all patients (100%) enrolled during hospitalization with significantly increased TBIL (56 ~ 300 μmol/L) dominated by DBIL and a mild-moderate elevated level of ALT (79 ~ 647 U/L) and AST (91 ~ 498 U/L). Raised uric acid (535 ± 85 μmol/L) was found in all patients (100%) and blood glucose decreased in 6 patients (46%) with a minimum of 2.4 μmol/L. Moreover, the main maternal complications were different degrees of coagulopathy (13/13, 100%), followed by acute renal dysfunction (10/13, 77%). In this study, a total of 12 of the 13 patients received abdominal ultrasound examination, among which five individuals fulfilled the ultrasonic characteristics of fatty liver, suggesting a diagnostic coincidence rate of 42%. It indicates that imaging examination is helpful for the diagnosis of AFLP.

**Table 2:** The laboratory results and imageological examinations of AFLP patients (n = 13)

Laboratory findings	Range	Mean ± SD
Age (years)	23–39	29.2 ± 5.1
Onset time (weeks)	32–38 + 6	34 + 5 ± 1.8
ALT (U/L)	79–647	324 ± 212
AST (U/L)	91–498	252 ± 156
TBIL (μmol/L)	56–300	143 ± 59
DBIL (μmol/L)	44–228	92.7 ± 51.5
Albumin (g/L)	22.8–37.0	27.6 ± 3.9
PT (s)	13.3–44.2	22.1 ± 9.1
INR	1.15–3.57	1.9 ± 0.7
Uric acid (μmol/L)	423–603	535 ± 85
Blood glucose (mmol/L)	2.4–9.87	4.9 ± 2.2
Cr (μmol/L)	126–286	186 ± 46.6
WBC count (×10^9^/L)	10.2–33.36	18.3 ± 6.4

AFLP: acute fatty liver of pregnancy; ALT: serum alanine aminotransferase; AST: serum aspartate aminotransferase; TBIL: total bilirubin; DBIL: direct bilirubin; PT: prothrombin time; INR: international normalized ratio; Cr: serum creatinine; WBC: white blood cell

### Treatment and outcome of the AFLP patients and their infants

Thirteen patients were definitively diagnosed with AFLP 0–2 days after the visit. Once the diagnosis was made, the pregnancy was terminated on the same day or next day. One (7%) maternal death occurred and all neonates (100%) survived delivery. The newborns delivered were all single births, including 8 boys, 5 girls, 10 premature infants and 3 full-term infants (Table 1). Plasma exchange was performed in 2 patients (15%) after termination of pregnancy, and 5 patients (38%) were transfused with blood products. Most patients had a good prognosis as a result of timely visit, while case 1 and case 2 unfortunately suffered from serious complications for delaying in seeking health care. In case 1 the pregnant women developed disseminated intravascular coagulation (DIC), intraperitoneal hemorrhage, acute pulmonary edema, multiple organ dysfunction and hemorrhagic shock at admission and finally discharged after a comprehensive treatment including cesarean section, blood transfusion, liver protection, anti-infection and blood purification. In case 2 the patient came to see the doctor at 10 days from the disease onset. She had severe liver injury and coagulation dysfunction on admission. Hence, termination of pregnancy was performed by cesarean delivery within 24 h after admission. However, the disease progressed rapidly. She developed abdominal hemorrhage and DIC, a serious life-threatening event, thus uterine artery embolization was immediately performed. Unfortunately, the patient was unsuccessfully resuscitated and died due to hemorrhagic shock.

### Biopsy pathology of the liver in the AFLP patients

**Fig. 1:**
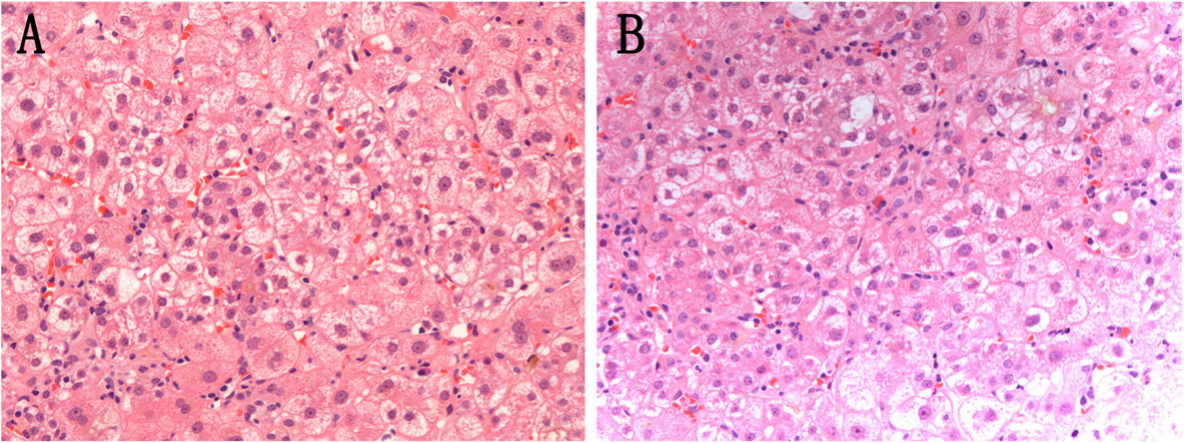
Biopsy pathology of the liver in the AFLP patients. Microvesicular steatosis of hepatocytes was observed in the liver specimens of the patients in case 8 (Fig. 1a) and case 10 (Fig. 1b), respectively (hematoxylin-eosin staining, magnification 200×)

Due to abnormal coagulation function of AFLP patients, liver biopsy is at high risk. In this study, 2 patients (case 8 and case 10) underwent liver biopsy guided by B-ultrasound 16 days and 23 days postpartum during the recovery period, respectively. The main pathological feature is characterized by microvesicular hepatic steatosis in Fig. 1, which meets the gold standard for the diagnosis of AFLP [9]. Other patients and their family members had concerns about the safety of liver biopsy and found it difficult to accept the invasive examination.

### Genetic test results in five patients and one newborn with high-throughput methods

Many foreign studies have pointed out that the development of AFLP is related to genetic mutations in pregnant women or newborns. Mutations reported so far are common defects in enzymes involved in fatty acid oxidation, such as LCHAD, CPTl, MCAD or SCAD [7, 10]. Combined with the notion that the diagnostic gold standard of AFLP is microvesicular hepatic steatosis, we speculated that any genetic defects affecting maternal or fetal fatty acid metabolism and resulting in the accumulation of fatty acid metabolites, may cause severe maternal liver damage, thus leading to AFLP. Therefore, we performed whole-exome sequencing in five patients and one newborn with high-throughput methods, hoping to explore gene mutations in Chinese AFLP patients at the gene level. We found that none of the five patients had mutations in the enzyme involved in fatty acid metabolism. In addition, whole-exome sequencing of peripheral blood in the newborn infant of his mother with AFLP still showed no related gene mutations in fatty acid oxidation.

## Discussion

Previous researches have shown that AFLP typically occurs during the third trimester of pregnancy, especially after 30 weeks of gestation. Also, there are rare cases occurring as early as 22 weeks of gestation [11–13]. Consistent with previous literature reports, all of 13 AFLP patients in this study have an onset in late pregnancy, with an average time to onset of 34 + 5 weeks. Besides, it was reported that multiple pregnancy, male fetus and co-existing other gestational liver diseases (e.g. HELLP, preeclampsia) are identified as risk factors for the incidence of AFLP [7]. As with the above, we found that there were three patients accompanied with preeclampsia and one patient accompanied with HELLP syndrome.

In our study, the main symptoms included jaundice, nausea, vomiting and abdominal pain in all pregnant women. However, all these manifestations were nonspecific. Patients were mainly hospitalized for further treatment about abnormal liver function. It is estimated that up to 3% of all pregnancies are complicated by liver disorders, which is broadly divided into 3 different groups: co-incident liver disease (most commonly viral hepatitis), pre-existing liver disease (such as viral hepatitis, Wilson’s disease and autoimmune liver diseases), and diseases unique to pregnancy [14]. A timely and accurate diagnosis to distinguish them is required so as to facilitate appropriate management, even sometimes urgent delivery is necessary if at the severe end of the spectrum. The timing of the onset of disease has been considered as a diagnostic information. Besides, clinical manifestations and laboratory examinations serve as the extremely significant basis to distinguish other gestational liver diseases. In this study, 13 patients mainly presented in late pregnancy with significant liver injury, coagulation dysfunction, acute renal injury, hypoglycemia and fatty liver, which meets the diagnosis of AFLP. Currently, the diagnosis of AFLP is based mainly on the Swansea criteria internationally. The Swansea criteria has a high diagnostic accuracy for the timely identification of AFLP patients so as to facilitate early intervention. Although liver biopsy is the gold standard for the diagnosis of AFLP, it is not routinely performed due to obvious coagulation abnormalities in patients and the invasiveness and complications of this procedure. Liver biopsy was often performed in patients for the etiology of unexplained liver injury or at postpartum.

To terminate the pregnancy as early as possible is the utmost importance for the treatment of AFLP, in which cesarean delivery is the first choice of mode of delivery. Delayed termination of pregnancy caused by missed diagnosis, misdiagnosis, or conservative treatment may lead to rapid progression of the disease, which could make the condition worse and hard to treat. In our study, these patients visited our hospital within zero to thirty days after disease onset, with average 8.3 days before the visit. A definitive diagnosis can be made 0–2 days after the visit. Once the diagnosis was made, the delivery was performed on the same day or next day. With increasing emphasis on health, most of patients were admitted in time due to abdominal discomfort, resulting in mild conditions or no serious complications. After a combined treatment including early termination of pregnancy, protection of liver and kidney function, blood transfusion, anti-infection and supportive care, overall patients have a short hospital stay and an improved prognosis. However, serious complications-abdominal hemorrhage and hemorrhagic shock caused by DIC occurred in two pregnant women due to a long time between the visit time and the onset time (10 days or 20 days). One patient was successfully rescued with great difficulty, while the other died after unsuccessful rescue. These cases further emphasize the importance of early visit, early diagnosis and early cesarean section.

Until recently, the etiology of AFLP has not been fully elucidated. This is partly due to the lack of knowledge about the disease and extremely low incidence of AFLP. Hence, few studies on this aspect have been done. Based on current researches, impaired beta oxidation of fatty acids in mother or fetus, particularly defects in the enzyme long-chain 3-hydroxyacyl-CoA dehydrogenase (LCHAD), which breaks down long-chain fatty acids in the liver, have been suggested to link to the development of maternal AFLP [15–17]. Ibdah’s study revealed that mothers of neonates with LCHAD deficiency have a 79% chance of developing AFLP or HELLP syndrome. Meanwhile, approximately 20% of neonates born to mothers with AFLP have been reported to have defects in β-oxidation due to a genetic mutation of LCHAD [18]. Among these, G1528C (E474Q) mutation in LCHAD gene is the most common molecular basis for AFLP. Ibdah et al. showed that G1528C mutation is the most common pathogenic mutation site in Western patients with LCHAD defect, and the population carrying rate is 1/175–680, which has become the most common fatty acid oxidation defect disease. Another study detected 3 cases of infants with LCHAD defect and found that 2 cases were homozygous for G1528C mutation and 1 case was compound heterozygote. The mothers of the 3 infants all developed AFLP or HELLP syndrome during pregnancy, and the G1528C mutation was inherited from the mother [15]. However, the LCHAD defect may be ethnically diverse. According to Zhu Jinming’s investigation in China, G1528C mutation was not found in cord blood samples of 1200 normal Chinese Han parturients [19]. Besides, Jiang Peiru detected the G1528C in cord blood samples of 12 groups of AFLP maternal and their newborn by Sanger sequencing and found no gene mutation [20]. Moreover, in a French study, G1528C and C1132T mutations in the LCHAD gene were not found in 14 pregnant women diagnosed with AFLP by histopathological examination [21]. Since AFLP is reported to be closely related to genetic background, it is suggested that there may be other gene mutation sites in Chinese population. In addition, other studies have pointed out that as long as gene defects induce dysfunction of maternal or fetal fatty acid metabolism, such as CPTl, MCAD and SCAD, it will promote accumulation of fatty acid metabolites and further cause damage to maternal liver, thus leading to AFLP [22–24]. Hence, the genetic background associated with the onset of AFLP was performed by high-throughput sequencing for the first time. To our surprise, whole-exome sequencing showed no gene mutation involved in fatty acid oxidation in the peripheral blood of five patients with AFLP and one newborn infant of his mother with AFLP. In addition, mutations in other genes known to predispose for the development of non-alcoholic fatty liver disease, e.g. PPARs, FXR, PNPLA3 weren’t seen in our study. On one hand, the sample size should be expanded for further verification. On the other hand, it suggests that there may be other pathogenic factors except genetic mutations, such as specific physiological changes during pregnancy, metabolic factors, etc., which need further exploration in the future.

## Conclusions

In summary, early visit, early detection, early termination of pregnancy and multidisciplinary comprehensive treatment are the key factors to improve the prognosis of AFLP patients. Contrary to common genetic mutant sites in Western people with AFLP, to our knowledge, although limited size of study, this report is the first to present the lack of common mutation involved in fatty acid oxidation in Chinese patients with AFLP via whole-exome sequencing. However, further studies are needed with larger and more varied samples to validate the conclusion.

## Supplementary Information

**Additional file 1.** General characteristics of the 13 cases of AFLP.

**Additional file 2.** Laboratory findings and imageological examinations of AFLP patients.

**Additional file 3.** The mutation sites filtered and annotated using GATK variant filtration and ANNOVAR in five AFLP patients.

## Data Availability

The datasets used and/or analysed during the current study are available from the corresponding author on reasonable request.

## Abbreviations

AFLP: acute fatty liver of pregnancy
WES: whole-exome sequencing
ACMG: American College of Medical Genetics and Genomics
ALT: serum alanine aminotransferase
AST: serum aspartate aminotransferase
TBIL: total bilirubin
DBIL: direct bilirubin
PT: prothrombin time
APTT: activated partial prothrombin time
WBC: white blood cell
Cr: serum creatinine
DIC: disseminated intravascular coagulation
LCHAD: long-chain 3-hydroxyacyl-CoA dehydrogenase
