# Supplementary material for: Analysis of clinical characteristics and genetic testing in patients with acute fatty liver of pregnancy: a retrospective study
Source: BMC Pregnancy Childbirth. 2021 Sep 8;21:617. doi: 10.1186/s12884-021-04095-8 (PMC8428114; doi:10.1186/s12884-021-04095-8)
Supplement: Supplementary file 1 — Additional file 1. General characteristics of the 13 cases of AFLP. [file 12884_2021_4095_MOESM1_ESM.docx]

**Additional file 1.** General characteristics of the 13 cases of AFLP.

| Case | Age (years) | Gravida | Chief symptoms | Delivery  mode | Onset time (weeks) | Visit time  (weeks) | | Diagnosis time  (weeks) | | Delivery time  (weeks) | Comorbidities | Maternal outcome | Perinatal outcome | Fetal sex |
| --- | --- | --- | --- | --- | --- | --- | --- | --- | --- | --- | --- | --- | --- | --- |
| 1 | 31 | multigravida | FAT/JAU | CS | 32+1 | 35 | 35+2 | | 35+2 | | HELLP;  preeclampsia | survival | survival | F |
| 2 | 26 | primigravida | JAU | CS | 35+4 | 37 | 37 | | 37+1 | | none | death | survival | M |
| 3 | 38 | multigravida | JAU/HEA | CS | 32 | 33+3 | 33+4 | | 33+4 | | preeclampsia | survival | survival | M |
| 4 | 31 | multigravida | JAU | CS | 38+1 | 38+6 | 38+6 | | 38+6 | | none | survival | survival | F |
| 5 | 23 | primigravida | FAT/NAU/VOM | V | 38+6 | 38+6 | 38+6 | | 38+6 | | none | survival | survival | M |
| 6 | 28 | primigravida | FAT | CS | 35+3 | 36+3 | 36+5 | | 36+5 | | none | survival | survival | M |
| 7 | 31 | multigravida | FAT/JAU | CS | 32 | 36+2 | 36+4 | | 36+4 | | none | survival | survival | F |
| 8 | 28 | primigravida | FAT/NAU/  JAU | V | 35+3 | 36+2 | 36+2 | | 36+2 | | none | survival | survival | F |
| 9 | 30 | primigravida | JAU | CS | 36 | 36+3 | 36+3 | | 36+4 | | none | survival | survival | M |
| 10 | 39 | multigravida | FAT/VOM/  JAU/ABD | CS | 34+2 | 35 | 35 | | 35 | | none | survival | survival | M |
| 11 | 23 | primigravida | NAU | CS | 33+5 | 34 | 34+2 | | 34+3 | | none | survival | survival | F |
| 12 | 28 | primigravida | FAT/NAU/  JAU/HEA | CS | 34 | 34+5 | 34+6 | | 34+6 | | preeclampsia | survival | survival | M |
| 13 | 23 | primigravida | FAT/NAU | CS | 35 | 35+5 | 35+5 | | 35+5 | | none | survival | survival | M |

FAT: fatigue; JAU: jaundice; HEA: headache; NAU: nausea; VOM: vomiting; ABD: abdominal pain; CS: caesarean section; V: vaginal delivery; none: no comorbidities; F: female; M: male.
