# Supplementary material for: Analysis of clinical characteristics and genetic testing in patients with acute fatty liver of pregnancy: a retrospective study
Source: BMC Pregnancy Childbirth. 2021 Sep 8;21:617. doi: 10.1186/s12884-021-04095-8 (PMC8428114; doi:10.1186/s12884-021-04095-8)
Supplement: Supplementary file 2 — Additional file 2. Laboratory findings and imageological examinations of AFLP patients. [file 12884_2021_4095_MOESM2_ESM.docx]

**Additional file 2.** Laboratory findings and [imageological examination](http://dict.youdao.com/w/imageological%20examination/#keyfrom=E2Ctranslation)s of AFLP patients.

| Case | ALT  (U/L) | AST  (U/L) | TBIL  (μmol/L) | DBIL  (μmol/L) | PT  (s) | APTT  (s) | WBC count  (×10^9^/L) | Uric acid  (μmol/L) | Blood glucose  (mmol/L) | Cr  (μmol/L) | Abdominal ultrasound | Liver biopsy | Genetic test |
| --- | --- | --- | --- | --- | --- | --- | --- | --- | --- | --- | --- | --- | --- |
| 1 | 79 | 119 | 300 | 228 | 34.9 | >240 | 13.4 | 499 | 3.3 | 165 | fatty liver;  ascites | / | yes |
| 2 | 122 | 124 | 168 | 130 | 44.2 | >240 | 12.6 | 423 | 8.8 | 213 | normal | / | yes |
| 3 | 127 | 91 | 127 | 92 | 13.3 | 47.4 | 10.2 | 527 | 5.52 | 128 | fatty liver;  ascites | / | / |
| 4 | 280 | 341 | 108 | 76 | 15.4 | 40.1 | 25.8 | 516 | 5.9 | 165 | normal | / | / |
| 5 | 599 | 498 | 168 | 57 | 21.4 | 46.6 | 19.1 | 756 | 2.4 | 286 | fatty liver | / | / |
| 6 | 179 | 184 | 102 | 56 | 19.7 | 46.1 | 18.2 | 586 | 3.7 | 217 | / | / | / |
| 7 | 338 | 120 | 108 | 62 | 17.0 | 59.0 | 23.4 | 494 | 5.1 | 215 | normal | / | yes |
| 8 | 83 | 92 | 175 | 140 | 29.5 | 62.1 | 33.36 | 513 | 9.87 | 234 | fatty liver | microvesicular steatosis | yes |
| 9 | 647 | 482 | 98 | 78 | 18.3 | 35.9 | 12.6 | 444 | 3.03 | 126 | normal | / | / |
| 10 | 349 | 226 | 154 | 63 | 17.7 | 55.7 | 21.7 | 489 | 3.15 | 152 | ascites | microvesicular steatosis | / |
| 11 | 257 | 178 | 56 | 54 | 14.1 | 40.6 | 16.8 | 513 | 3.85 | 139 | normal | / | yes |
| 12 | 619 | 364 | 133 | 44 | 17.1 | 41.5 | 17.0 | 595 | 4.9 | 187 | normal | / | / |
| 13 | 533 | 460 | 162 | 125 | 25 | 50 | 14.3 | 603 | 4.66 | 187 | fatty liver | / | yes* |

ALT: serum alanine aminotransferase; AST: serum aspartate aminotransferase; TBIL: total bilirubin; DBIL: direct bilirubin; PT: prothrombin time; APTT: activated partial prothrombin time; WBC: white blood cell; Cr: serum creatinine; /: not performed. yes*: Genetic test is performed in this newborn infant of his mother with AFLP.
